# Capitalizing on transcriptome profiling to optimize and identify targets for promoting early murine folliculogenesis in vitro

**DOI:** 10.1038/s41598-021-92036-y

**Published:** 2021-06-15

**Authors:** Andrea Jones, Beatriz Peñalver Bernabé, Vasantha Padmanabhan, Jun Li, Ariella Shikanov

**Affiliations:** 1 Department of Biomedical Engineering, University of Michigan, 2126 Lurie Biomedical Engineering, 1101 Beal Avenue, Ann Arbor, MI 48109 USA; 2 Department of Bioengineering, College of Medicine, University of Illinois Chicago, Chicago, IL 60607 USA; 3 Department of Human Genetics, University of Michigan, Ann Arbor, MI 48109 USA; 4 Department of Computational Medicine and Bioinformatics, University of Michigan, Ann Arbor, MI 48109 USA; 5 Department of Pediatrics and Communicable Diseases, University of Michigan, Ann Arbor, MI 48109 USA; 6 Department of Obstetrics and Gynecology, University of Michigan, 2126 Lurie Biomedical Engineering, 1101 Beal Avenue, Ann Arbor, MI 48109 USA; 7 Department of Molecular and Integrative Physiology, University of Michigan, Ann Arbor, MI 48109 USA; 8 Department of Macromolecular Science and Engineering, University of Michigan, 2126 Lurie Biomedical Engineering, 1101 Beal Avenue, Ann Arbor, MI 48109 USA

**Keywords:** Biotechnology, Computational biology and bioinformatics, Engineering

## Abstract

In vitro ovarian follicle culture is an active area of research towards providing fertility options for survivors of childhood cancer. Late-stage murine follicles (multilayer secondary and onwards) can be cultured successfully to maturity to obtain a meiotically competent oocyte for fertilization, but primordial and primary follicles usually die in culture because many key components of early follicle development are still unknown and difficult to mimic in vitro. To engineer a biomimetic three-dimensional culture system with high efficacy and reproducibility for the clinic, detailed mechanisms of early folliculogenesis must be uncovered. Previous studies have shown that primary murine follicles co-cultured in groups, in contrast to single follicles cultured in isolation, can reach preovulatory size and produce competent oocytes, but the factors accounting for the synergy of follicle co-culture are still unknown. To probe the underlying mechanisms of successful follicle co-culture, we conducted a time-course experiment for murine follicles encapsulated in 0.3% alginate hydrogels and compared between two conditions: groups of 5 (5X) versus groups of 10 (10X). For every 2 days during the course of 12 days, follicles were dissociated and somatic cells were isolated for microarray-based gene expression analysis (n = 380 follicles for 5X and n = 430 follicles for 10X). Gene activities in follicles co-cultured in larger groups (10X) had a distinct transcriptomic profile of key genes and pathways such as prolactin signaling and angiogenesis-related genes when compared to cells from follicles co-cultured in the smaller cohort (5X). To benchmark the results for follicles grown in culture, we compared our microarray data to data from murine follicles freshly isolated from the ovary at comparable stages of development previously published by Bernabé et al. Comparison of these datasets identified similarities and differences between folliculogenesis in the native microenvironment and the engineered in vitro system. A more detailed understanding of follicle growth in vitro will not only allow for better culture methods but also advance the field towards providing improved fertility options for survivors of childhood cancer.

## Introduction

Ovarian follicle growth is a complex biological process directed by local environmental cues, including hormones from the hypothalamic–pituitary–gonadal (HPG) axis. The follicle, comprised of the female gamete (oocyte) surrounded by somatic cells, is the functional unit of the ovary^1,2^. The non-renewable follicular reserve of a woman is gradually depleted over her reproductive lifespan as follicles activate, enter the growing pool, and eventually degenerate. Early follicle growth is believed to be independent of circulating systemic hormones and heavily relies on paracrine signaling within the follicular structure and local extrafollicular environment^2^. Numerous studies have investigated secreted factors that impact folliculogenesis, but much is still unknown about the signaling pathways and related cytokines that initiate primordial follicle activation and direct the transitions from primordial to primary and later secondary stages of folliculogenesis^3–8^.

Primordial and primary follicles comprise the majority of the ovarian reserve and are critical for individuals who face anti-cancer treatments, which are largely gonadotoxic and prematurely deplete the follicle reserve in female patients. Cryopreservation of eggs or embryos is the clinically approved fertility preservation option for post-pubertal individuals, but the hormones required for ovarian stimulation and egg retrieval can aggravate the pathogenesis of certain types of cancer, and patients with aggressive cancers cannot delay treatment^9^. Furthermore, ovarian stimulation and egg retrieval are not suitable for pre-pubertal girls. The significant limitations of current fertility preservation options for aforementioned patients and the recent increase in survival rates for most childhood cancers calls for development of reliable methods for growing and maturing healthy eggs from patients’ cryopreserved ovarian tissue^10^.

Three-dimensional (3D) hydrogel-based in vitro culture systems are commonly employed for murine ovarian follicle culture^11–14^. Follicles cultured in these matrices maintain their 3D structure and the cellular connections necessary for successful growth. Alginate, a polysaccharide derived from seaweed, has been widely implemented because of its mild gelation and tunable physical properties, which are important for follicle culture^10^. 3D culture in alginate has been shown to support larger, preantral follicles in vitro, however culture of small early-stage follicles remains challenging^15^. It has been hypothesized that the lack of progress in this area is due to the inability of current culture systems to supply the essential factors to individual primordial and primary follicles that would otherwise be provided through paracrine interactions in the native ovarian stroma and neighboring follicles in vivo^16^.

One option to address this challenge is to co-culture follicles with “feeder cells” such as murine embryonic fibroblasts (MEFs), stromal cells, ovarian mesenchymal cells, and granulosa cells that secrete factors to promote follicle growth and survival^17–20^. Alternatively, co-culture of primary murine follicles in groups has been shown to significantly improve growth and survival when compared to culture of individual primary follicles, with groups of 10 (10X) cultured together being more successful than groups of 5X^11^. However, the unknown identity of the secreted factors and inconsistency between different batches of cells or follicles limit the reliability and translational potential of these approaches. A recent study by our research group probed the secretome and transcriptome signatures of follicles that were co-cultured as 10X and 5X with a goal of identifying factors present in 10X that drive successful folliculogenesis^21^. Follicles cultured in groups of 10 had upregulated activity of key transcription factors as well as a unique secretory signature, revealing some of the biological processes and pathways essential for early follicle development. Specifically, using a targeted approach, this study determined that the mechano- and oxygen-responsive transcription factors, NF-κB and HIF1, were upregulated and correlated with the antrum formation in 10X but not 5X follicle cultures. Unsupervised transcriptomic analysis of later stages of follicular development^22–24^ suggests an unparalleled opportunity to profile global transcription activity of early stage follicles cultured in groups of 5 and 10 in a controlled in vitro environment. While follicles in the 5X condition survive and grow, they yield oocytes at a significant lower quality and quantity compared to those from the 10X condition. We hypothesized that unbiased transcriptome comparison of follicles grown in suboptimal (5X) and optimal (10X) environments will identify key mediators of successful early folliculogenesis. Here we performed microarray analysis of somatic cell samples from primary murine follicles co-cultured in 5X and 10X, each over a 12-day time course, to identify upstream regulators of follicle growth and maturation that contribute to the successful follicle culture in vitro and to further characterize the unique synergism of successful culture of follicles in larger groups. The analytical approaches undertaken sought to answer three key biological questions: (1) what temporal transcriptional signatures drive folliculogenesis in vitro, (2) what differentially regulated genes and pathways drive better survival, growth, and maturation in the 10X condition, and (3) how do the in vitro and in vivo follicular transcriptomes compare on a global scale.

## Results

### Maturation characteristics of longitudinal follicle co-culture experiments

**Figure 1:**
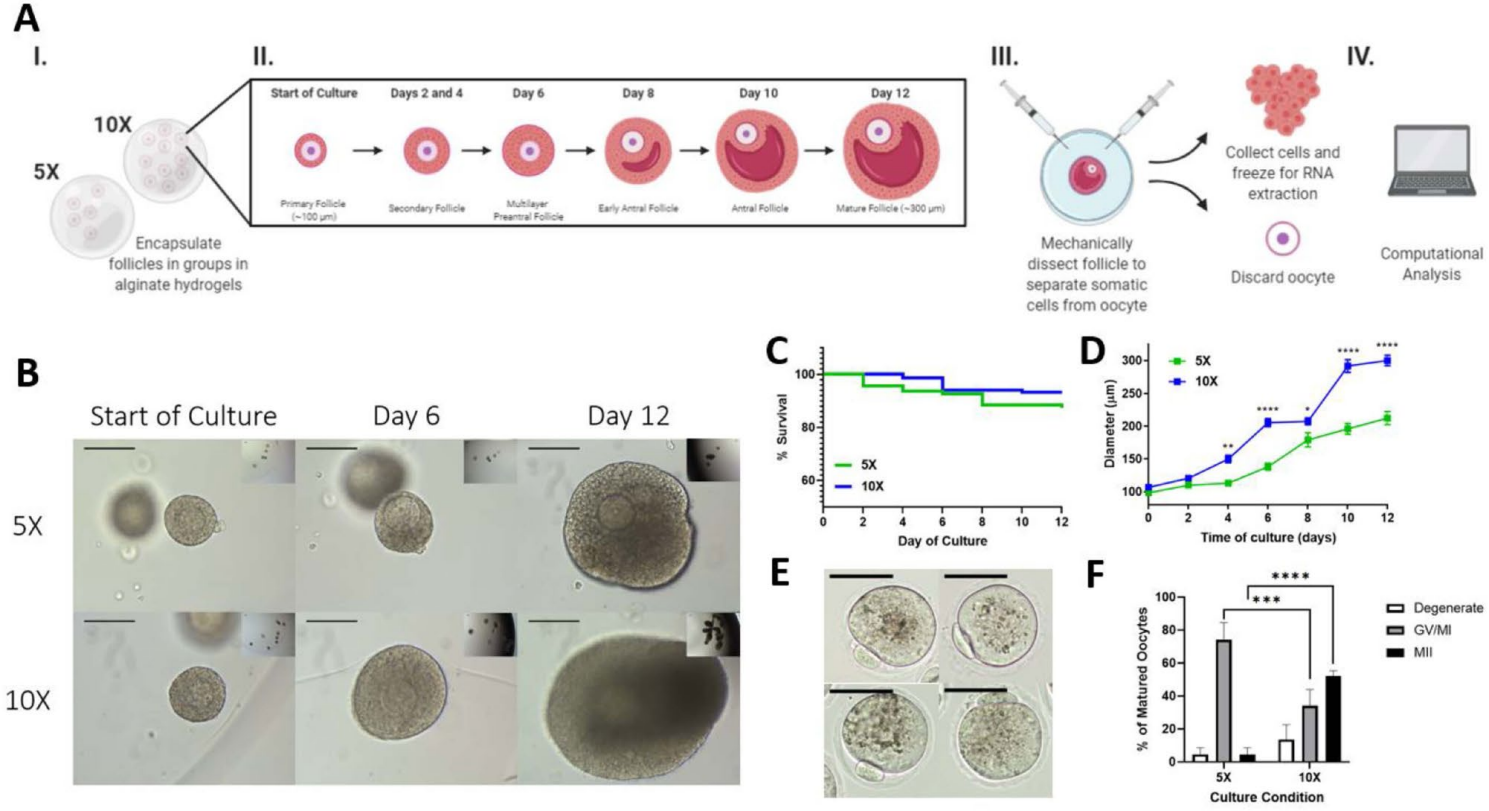
Ovarian follicles cultured in 10X have superior outcomes when compared to 5X culture. (**A**) Schematic describing the process of somatic cell isolation, beginning with primary follicles cultured in alginate hydrogels over 12 days, during which follicles are dissected at two-day intervals for cell collection, (**B**) bright field images of individual primary follicles encapsulated in alginate hydrogels in either groups of 5 or 10 (scale bar = 100 µm), (**C**) survival rates of follicles over duration of culture in 5X and 10X, (**D**) growth rates of follicles over duration of culture in 5X and 10X, and (**E**) metaphase II oocytes from follicles cultured in groups (scale bar = 50 µm), and (**F**) in vitro maturation rates for follicles cultured in 5X and 10X. N = 95 follicles for 5X and N = 140 for 10X in Fig. 2B–D. N = 39 follicles for 5X and N = 204 for 10X for Fig. 2F. *p < 0.05, **p < 0.01, ***p < 0.001, ****p < 0.0001. (**A**) was generated using Biorender (biorender.com).

Follicles encapsulated in alginate hydrogels in 5X and 10X conditions were maintained for 12 days (Fig. 1A). They grew at different rates, reaching a mean diameter of 212.36 ± 10.04 and 300.02 ± 8.00 µm (mean ± SEM) respectively (Fig. 1B, D), corroborating previous findings^11,21^. There were no statistical differences in follicle survival rate between 5 and 10X conditions (Fig. 1C). However, maturation of the follicles cultured in groups of 10 that reached a diameter ≥ 280 µm resulted in 50% of the oocytes resuming meiosis and reaching metaphase II stage, which was significantly greater (*p* < 0.0001) than the maturation rates of ~ 5% for oocytes from follicles cultured in 5X (Fig. 1E, F).

### Trajectories of the 5X and 10X transcriptomes during culture

**Figure 2:**
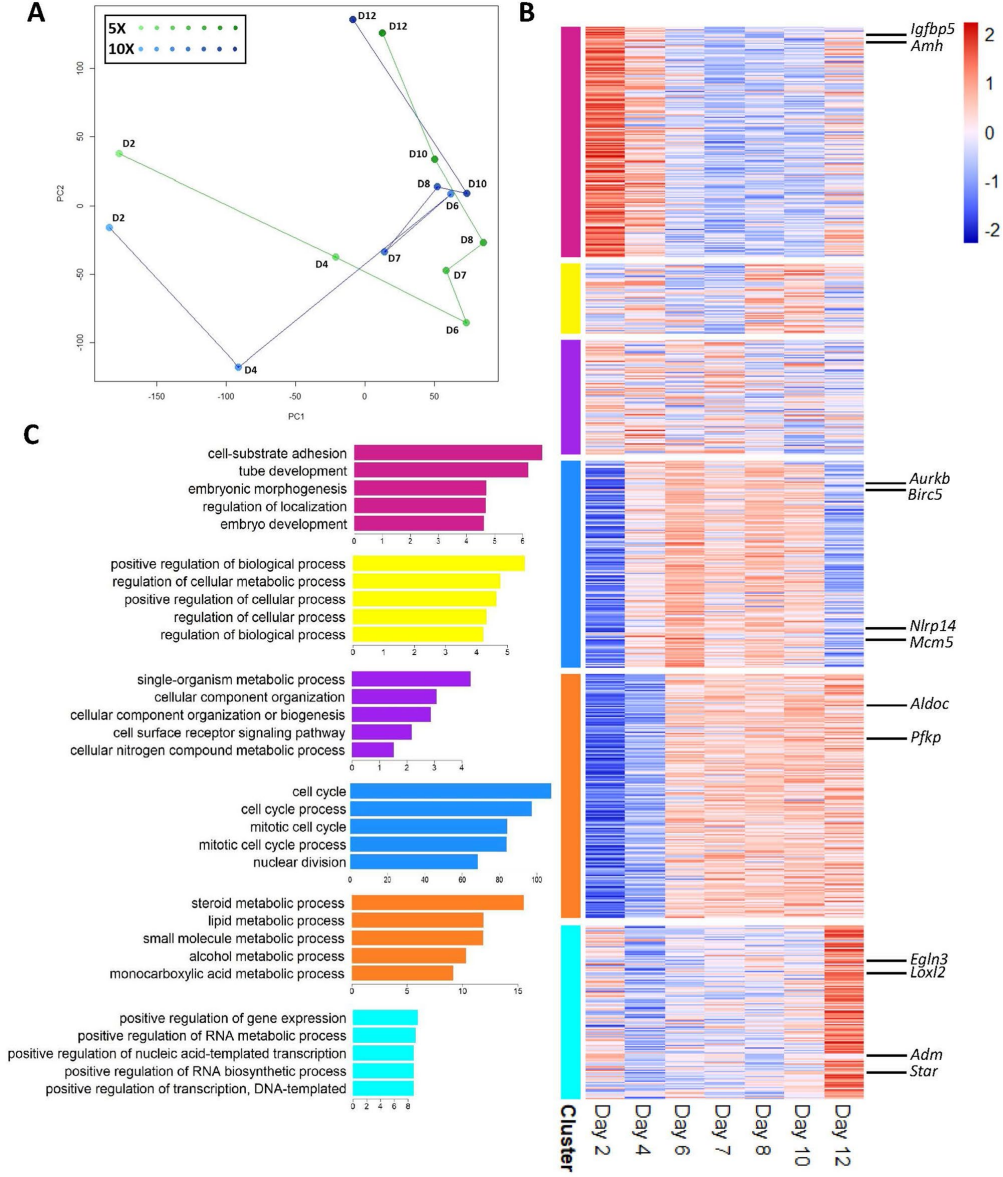
Somatic cells from ovarian follicles cultured in groups have dynamic temporal gene expression. (**A**) Principal component plot of the 14 microarray samples, (**B**) heatmap of standardized expression level for genes in the six clusters obtained from k-means analysis (k = 6), and (**C**) top five GO terms enriched for differentially expressed genes in each cluster from *LRPath* analysis (x-axis = − log(*p* Value)). (**B**) was generated using pheatmap (version 1.0.12)^62^ in R (version 3.6.9, https://www.r-project.org/)^55^. (**A**) and (**C**) were generated in R (version 3.6.9, https://www.r-project.org/)^55^.

We analyzed 14 RNA samples representing both experimental groups (5X and 10X) during a 7-point time course each, sampled at a regular interval of two days (Methods). Each sample contained 40–70 follicles (Supplementary Table S1). While all other samples were collected on even days of culture (2, 4, 6, 8, 10, 12), we included the Day 7 samples because of the dramatic phenotypic changes observed between Days 6 and 8 in vitro, including antrum formation and cell proliferation. By adding this additional time point we hoped to capture transcriptional changes at this crucial point in development. After data normalization and filtering for the most variable 13,313 genes, the transcriptional data for the 14 samples were subjected to an unsupervised principal component analysis (PCA) (Fig. 2A). In the principal component (PC) space, both 5X and 10X samples travel along PC-1 initially, and then along PC-2 towards the end of culture, reflecting transcriptional similarities between the two culture conditions. The paths of 5X and 10X diverge most dramatically at Day 4 (Fig. 2A). The trajectories seen in the PC space guided our approach for evaluating differences between the 5X and 10X conditions, where 5X appears to be slower to progress through the middle stage when compared to 10X (Fig. 2A). With this in mind, our comparisons of the 5X and 10X culture systems (next section) sought to identify genes and pathways that lag behind in 5X when compared to 10X.

### Genes and pathways with distinct temporal patterns

The expression levels for the 13,313 genes were standardized for each gene and shown for the 14 samples in Figure S2. To find groups of genes with shared dynamic patterns during the 7-point time series, without accounting for the 5X versus 10X condition but instead evaluating the in vitro microenvironment as a whole, we applied k-means clustering (k = 6) to define gene clusters according to their expression levels in the 14 independent samples (for 5X and 10X, 7 from each condition). In Fig. 2B, the 5X and 10X series are averaged, resulting in a consensus 7-point profile, ordered by time and separated by the six gene clusters. For genes within each cluster we performed differential expression (DE) analysis to identify those with the most salient changes, with the comparison groups defined for each cluster based on its own patterns (Supplementary Table S1). For example, for the 4th gene cluster (indicated in blue in Fig. 2B), DE analysis was made by comparing the middle five time points as the High group with the first and last points combined as the Low group. Among the six clusters, four had strongly concerted patterns of up- and down-regulation; while the other two, the 2nd and 3rd cluster, had undefined oscillatory changes throughout the culture. We therefore focused our DE results interpretation on the four clusters with distinct dynamic changes, at both the gene level and the pathway level. To identify differentially-expressed genes, we analyzed each gene using *limma*, and ranked genes within each cluster by log-fold change (logFC) (Supplemental Table S2). For pathway analysis, DE-ranked genes in a cluster were subjected to gene ontology (GO) analysis using *LRPath* and *GOrilla*, with a focus on Biological Process GO terms (Methods). The first cluster (n = 1826 genes, labeled with magenta) contains “early activators”: genes with high expression at the beginning of culture, and are downregulated as time in culture progresses (Fig. 2B). Genes of interest in the top 100 genes according to logFC in this cluster include *Amh* (coding for anti-Müllerian hormone) and *Igfbp5* (coding for insulin-like growth factor-binding peptide 5), both of which have known roles in granulosa cell survival and proliferation (Supplemental Figure S3)^25,26^. The top GO terms for this cluster include “cell-substrate adhesion” (GO:0031589, 53 genes), “embryonic morphogenesis” (GO:0048598, 71 genes), and “embryo development” (GO:009790, 116 genes) (Fig. 2C). Genes of interest within the cell-substrate adhesion GO term include genes coding for angiopoietin 1 and 2, collagen types I and VIII, and genes coding for laminin and matrix metalloproteases (Supplemental Dataset S1). *GOrilla* analysis of this cluster also showed enrichment for terms associated with cell adhesion and migration, cell proliferation, and signal transduction and metabolic processes (Supplemental Dataset S2). The fourth cluster (n = 1630 genes, shown in blue in Fig. 2B) mainly contains genes with delayed upregulation on Day 4 and a decline in regulation between Days 10 and 12. Genes of interest with high logFC in this cluster include *Aurkb*, *Birc5*, and *Mcm5*, which code for aurora kinase B, baculoviral inhibitor of apoptosis repeat-containing 5 (BIRC5), and the protein MCM5 respectively, all of which have known roles in the cell cycle and apoptosis, and *Nlrp14*, coding for the protein NOD-like receptor family pyrin domain containing 4 (NLRP14), which has been studied in relation to primordial follicle survival and may have other roles in later stages of folliculogenesis^27,28^. All five top GO terms for this cluster were associated with cell cycle activity: “cell cycle” (GO:0007049, 267 genes), “cell cycle process” (GO:0022402, 217 genes), “mitotic cell cycle” (GO:0000278, 173 genes), “mitotic cell cycle process” (GO:1903047, 165 genes), and “nuclear division” (GO:0000280, 147 genes) (Supplemental Dataset S1). This period of upregulation from Days 4 to 10 reflects the proliferation of granulosa cells during follicular expansion, which ceases once the follicle has reached a terminal size (Fig. 2C). *GOrilla* analysis corroborated these findings, also reporting enrichment for terms related to DNA replication and the cell cycle (Supplemental Dataset S2). The fifth cluster (n = 1928 genes, labeled in orange), was comprised of genes with delayed activation, as they were upregulated starting on Day 6 (Fig. 2B). Genes of interest within this cluster included glycolysis-related genes like *Aldoc* (coding for aldolase C) and *Pfkp* (coding for phosphofructokinase), noteworthy because granulosa cells are known to have highly glycolytic metabolic processes often regulated by oocyte-secreted factors^29^. The top GO terms for this cluster from *LRPath* were all related to metabolism: “steroid metabolic process” (GO:0008202, 54 genes), “lipid metabolic process” (GO:0006629, 146 genes), “small molecule metabolic process” (GO:0044281, 201 genes), “alcohol metabolic process” (GO:0006066, 50 genes), and “monocarboxylic acid metabolic process” (GO:0032787, 75 genes) (Fig. 2C) (Supplemental Dataset S1). The last cluster (n = 1378 genes, labeled in cyan in Fig. 2B) was comprised of genes with late activation in the final days of culture. Genes of interest in this cluster included *Adm* (coding for adrenomedullin) and *Star* (coding for steroidogenic acute regulatory protein), both of which drive steroidogenesis in the follicle, and *Egln3* (coding for Egl-9 family hypoxia inducible factor 3) and *Loxl2* (coding for lysyl oxidase like 2), which are both hypoxia-related^30,31^. Top GO terms from *LRPath* and *GOrilla* for this cluster included “positive regulation of gene expression” (GO:0010628, 86 genes) and “positive regulation of transcription, DNA-templated” (GO:0045893, 66 genes) (Fig. 2C) (Supplemental Dataset S1, Supplemental Dataset S2).

### Comparison between 5 and 10X transcriptome trajectories

**Figure 3:**
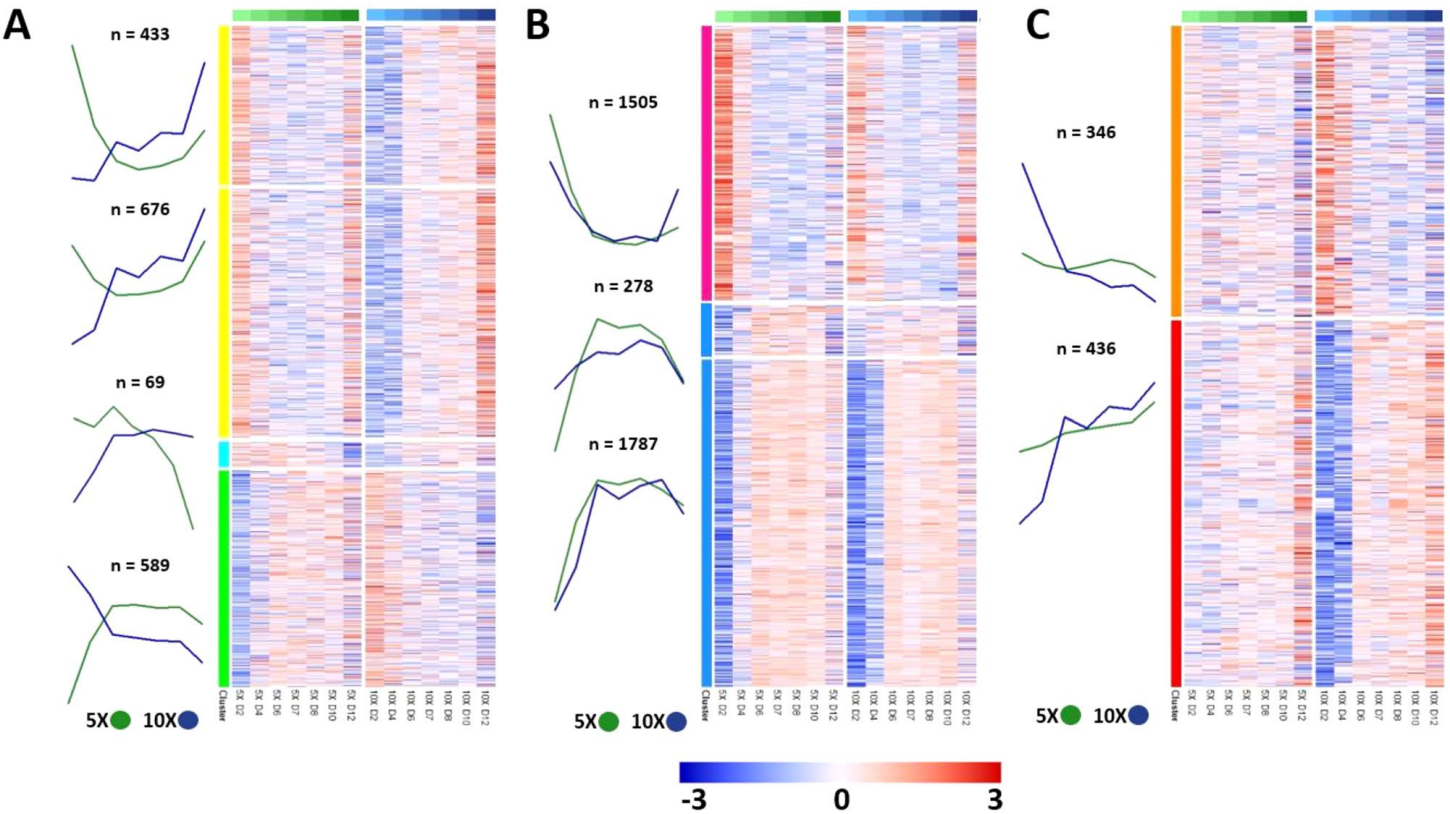
Somatic cells from follicles cultured in 5X versus 10X have distinct gene expression profiles in nine gene subsets. Mean expression plots with gene number and per-gene heatmaps for (**A**) gene subsets with different expression in 5X versus 10X, (**B**) gene subsets with similar expression in 5X versus 10X, and (**C**) gene subsets with more dynamic expression in 10X versus 5X. All plots were generated using pheatmap (version 1.0.12)^62^ in R (version 3.6.9, https://www.r-project.org/)^55^.

While the results above focused on 5X-10X shared patterns, we also contrasted the 5X and 10X transcriptomes to identify genes and pathways with differing patterns of expression. The two datasets were filtered to select genes with dynamic temporal expression and high cohesion across the time series (Methods), thus eliminating genes with stochastic “spikes” in expression. The resulting set of 6,119 genes were clustered into nine subsets using a 3 × 3 self-organizing map^32^, then subjected to gene ontology analysis of each subset (Fig. 3). The gene subsets showed three general patterns of dynamic change: (1) genes with opposite temporal expression patterns between 5X versus 10X at the beginning, the end, or both (Fig. 3A), (2) genes with largely similar temporal expression patterns in 5X and 10X (Fig. 3B), and (3) genes with dynamic expression in the 10X condition in contrast to a more static expression in the 5X condition (Fig. 3C). These gene subsets were individually subjected to gene ontology analysis, with the exception of the top two subsets in Fig. 3A, which were merged into a single group due to their similar mean expression patterns.

In the top two subsets in Fig. 3A (marked in yellow) the gene transcription of follicles cultured in 5X followed a “U-shape” form, starting higher at Day 2 of the culture, dropping to the minimum after 4 days and climbing back up towards the end of the culture. In contrast, the gene transcription of follicles cultured in 10X continuously increased from Day 2 to Day 12. These genes include those related to MAPK signaling, prostaglandins, *Cyp11b1*, which is involved in the biosynthesis of cortisol, and *Star*, which is involved in progesterone synthesis in the follicle. The yellow subset showed enrichment for genes related to calcineurin-NFAT signaling (GO::0051534 and GO::0106057) (Supplemental Dataset S3). In the bottom subset (marked in green) the gene transcription of follicles cultured in 5X followed an inverse “U-shape” form, starting low at Day 2 of the culture, climbing to the maximum after 4 days and slightly decreasing towards the end of the culture. In contrast, the gene transcription of follicles cultured in 10X monotonically decreased from Day 2 to Day 12, following a similar trajectory to 5X in the last few days of the culture. The green subset included genes related to angiogenesis (angiogenins and angiopoietins), as well as Wnt signaling and was enriched for gene ontology terms on G protein-coupled receptor signaling (GO::0007186) and sensory perception of chemical stimulus (GO::0007606) (Supplemental Dataset S3). In the middle subset (cyan) the gene transcription of follicles cultured in 5X dramatically decreased since Day 4 of the culture and reached its lowest at the end of the culture. In contrast, the gene transcription of follicles cultured in 10X slowly increased from Day 2 to Day 12, presenting an opposite trajectory to 5X. The cyan subset included prostaglandins and was enriched for terms related to lactation (GO::1903489 and GO::1903487) and JAK/STAT signaling (GO::2000366) (Supplemental Dataset S3).

Among the genes with similar temporal expression in 5X and 10X (Fig. 3B), the magenta subset was enriched for gene ontology terms related to transmembrane transport and locomotion (GO::0034765, GO::0034762, and GO::0040012) (Supplemental Dataset S3). The two blue subsets in Fig. 3B were enriched for genes related to the cell cycle (GO::0007049, GO::0022402, GO::1903047, GO::0051301, and GO::0051726) (Supplemental Dataset S3).

The third group of genes with more dynamic expression in 10X compared to 5X (Fig. 3C) was enriched for terms related to G-protein coupled receptor activity (GO::0007186) and sensory perception of chemical stimulus (GO:: 0007606) in the orange subset, and terms related to protein modification by small protein conjugation (GO::0032446) and protein ubiquitination (GO::0016567) in the red subset (Supplemental Dataset S3).

### Comparisons of transcriptome data between freshly isolated and in vitro cultured follicles

**Figure 4:**
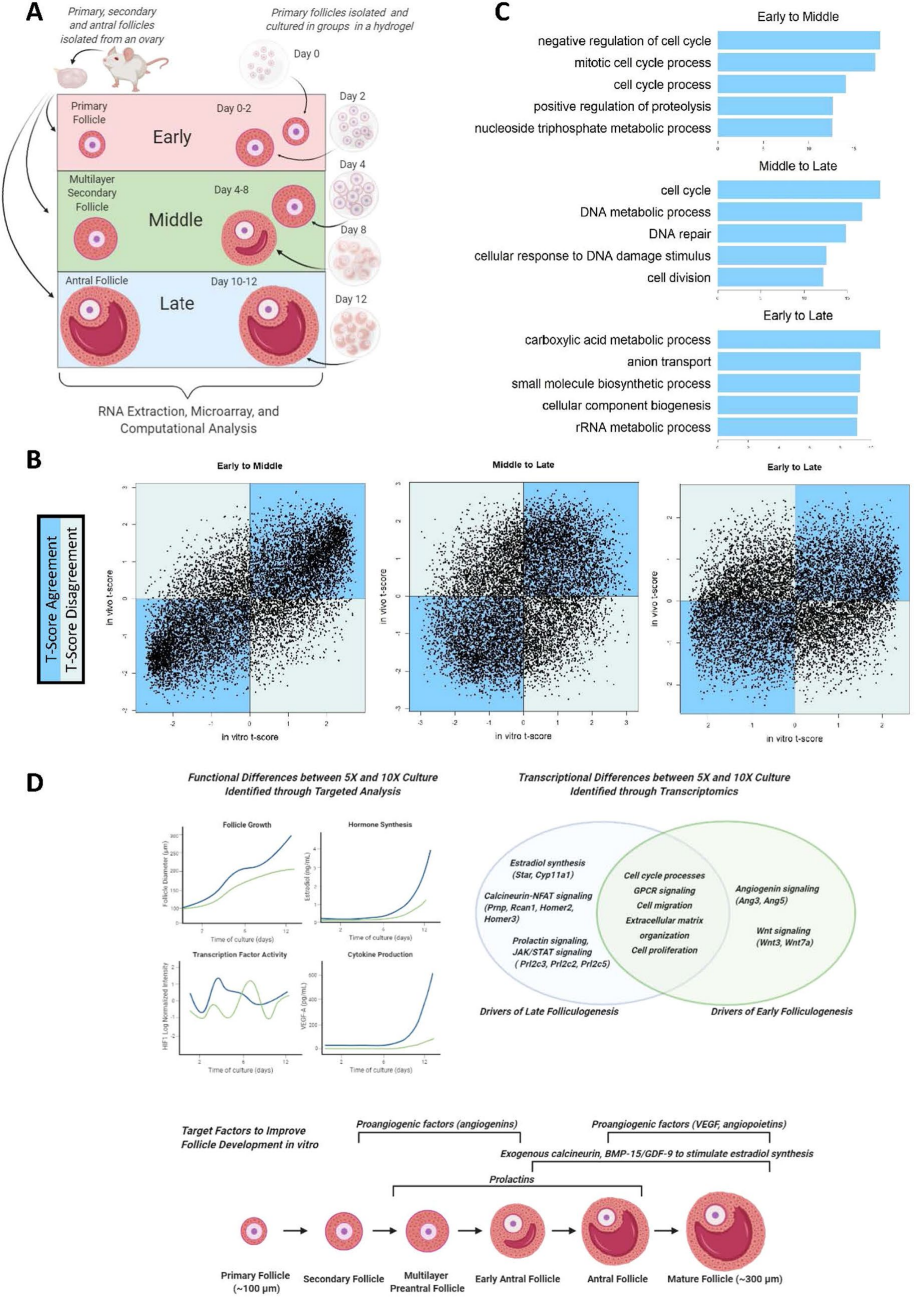
Ovarian follicles developing in vitro, compared to those developing in vivo, show significant transcriptional similarities and differences. (**A**) Schematic explaining methods for comparing in vivo dataset to 5X and 10X dataset, (**B**) t-score agreement plots (r = 0.704, 0.345, and 0.212 respectively), (**C**) top GO terms from *GOrilla* analysis, highlighting GO terms from the gene lists with shared t-scores signs between in vitro and in vivo, and (**D**) summary of study findings: previously described targeted analyses from Zhou et al. 2018, combined with transcriptome analysis, leads to identification of targets for improving in vitro follicle culture. (**A**) was generated using Biorender (biorender.com). (**B**) and (**C**) were generated using R (version 3.6.9, https://www.r-project.org/)^55^.

To compare the transcriptome profile of somatic cells from follicles co-cultured in vitro to those from freshly isolated follicles in vivo at comparable stages of development, we drew on previously published data available online on the Gene Expression Omnibus (GSE 97902)^33–35^. This published dataset was used to develop a metabolic model for murine ovarian follicles by updating the previously published dataset Mouse Recon 1 to include human homologues and key ovarian follicle development pathways that were not previously represented^33,36^. The dataset used during model development includes transcriptome data from the follicular structure as a whole (including the oocyte and somatic cells) at various stages of follicle development^33^. While the experimental approach used in collecting these samples inevitably differs from the methods we used, parallels can be drawn in follicular development as shown in Fig. 4A. To compare transcriptional profiles between the two datasets, the “Primary” data from GSE 97902 was considered equivalent to the in vitro Day 0 and Day 2 samples, and subsequently the “Multilayer Secondary” and “Antral” datasets from GSE 97902 were considered equivalent to Days 4 through 8 and Days 10 and 12 respectively (Fig. 4A). After normalization and filtering, 11,424 genes were shared between the two datasets. To compare temporal gene expression patterns between the in vitro and in vivo datasets, differential gene expression analysis was performed for each developmental transition (Early to Middle, Middle to Late, Early to Late) for the two datasets separately, where the gene expression values for the in vitro dataset were taken as the average gene expression between 5 and 10X. We employed this combined in vitro dataset instead of comparing the 5X and 10X datasets to the in vivo dataset individually to maximize the statistical power of the analysis and to draw conclusions about the in vivo versus in vitro microenvironments, keeping in mind that the in vivo microenvironment is highly complex and would be difficult to fully replicate in vitro. Because of the differences in sample collection between the two datasets, and the difference in microarray chip formats, we did not directly compare normalized expression values between the two datasets. Instead, the t-score for each gene from differential gene expression analysis was used to compare the changes in gene expression over analogous transitions (i.e. a gene with a positive t-score in both in vitro and in vivo datasets is one that went up in expression from Time A to Time B in both data subsets). The t-scores from the two datasets were compared and plotted in Fig. 4B. The most agreement between the two datasets was observed from the Early to Middle stages of follicle development (r = 0.704, *p* < 0.0001 for testing r = 0 using Fisher’s transformation). The Middle to Late and Early to Late comparisons showed less agreement (r = 0.345 and r = 0.212 respectively). The t-score agreement and disagreement gene lists were subjected to *GOrilla* analysis to identify GO term enrichment for each dataset and thus cellular processes and pathways that were similar and dissimilar when comparing the in vitro and in vivo datasets (Supplemental Dataset S4, Supplemental Image File Folder S3). In both Early ~ Middle and Middle ~ Late comparisons, GO terms associated with the cell cycle were enriched, and in the Early ~ Late comparison various metabolic processes were enriched within the matched gene lists, indicating that these processes behave similarly in the in vivo and in vitro settings (Fig. 4C).

## Discussion

### The somatic cell transcriptome from group follicle culture reflects dynamic expression of known and novel regulators of folliculogenesis

The group follicle culture system presented here and previously studied by Hornick et al. and Zhou et al. serves as a highly controlled, tunable platform for studying folliculogenesis with the goal of obtaining mature oocytes from cultured follicles^11,21^. Unlike follicles in vivo, where the oocyte’s maturation potential cannot be determined, the group follicle culture system has been characterized such that suboptimal and optimal culture conditions can be directly compared and samples can be taken from a controlled environment. Previous analyses of group follicle culture have identified 10X culture as superior to 5X with clear secretory and transcriptional distinctions between the two group sizes^11,21^. In vitro maturation of oocytes from follicles cultured in groups has also shown the advantage of 10X culture over 5X, which produced significantly fewer mature metaphase II oocytes (Fig. 1F). Using time-series transcriptomics starting at the primary stage in vitro, we have provided a holistic view of the dynamic processes involved in folliculogenesis. Understanding temporal expression of genes with known roles in folliculogenesis, coupled with identification of novel genes and pathways, can enhance optimization of current culture systems by providing a biological basis for supplementing culture media with additional factors identified through analysis of this dataset. Principal component analysis (PCA) of the 5X and 10X microarray data showed that the two systems followed similar trajectories through the reduced dimensional space, reflecting the inherent transcriptional similarities between the two group sizes (Fig. 2A). Later days of culture (6 through 10) were closely clustered, indicating transcriptional similarities between conditions and across these time points that are reflected phenotypically by follicular expansion and antrum formation seen in both group sizes.

Among the six clusters of genes shown in Fig. 2B, four had distinct temporal profiles. The cluster termed “early activators” (magenta) (Fig. 2B, C) showed enrichment for GO terms associated with cell-substrate adhesion, as well as survival and proliferation. This may reflect the follicular cells’ adjustment to the in vitro culture environment after removal from the ovary and encapsulation in a hydrogel, as well as the initial granulosa cell proliferation during the follicle’s transition from the primary to secondary stages. The genes *Amh* and *Amhr2*, which code respectively for anti-Müllerian hormone (AMH) and one of its receptors, belong to this early activator group in magenta (Fig. 2B, C). AMH has been thoroughly studied as an early regulator of folliculogenesis and is known to inhibit activation of primordial follicles. The early peak of expression and subsequent downregulation observed in our dataset agrees with the expression patterns reported in other studies^37–39^. Interestingly, the fifth cluster, termed “delayed activators” (orange), was enriched for terms related to steroidogenesis—this may reflect the follicle’s increased signaling activity as the antrum forms and the follicle becomes hormone-responsive (Fig. 2B, C). Both *Cyp17a1* and *Cyp19a1* belong to this cluster—the protein CYP17 is essential for androgen production within the ovary, and in turn, the gene *Cyp19a1* codes for the protein aromatase, which converts androgen into 17β-estradiol^40,41^. The delayed activation of both these genes in vitro reflects the timing of hormone production in the follicular structure, which does not occur until the preantral stages and beyond. The transcriptional activity of the transient fourth cluster (blue) peaked in the middle of culture (Fig. 2B) and was enriched for cell cycle-related genes which agrees with the rapid cell proliferation throughout follicle development in vivo (Fig. 2C). *Bmp15* was also assigned to the fourth cluster and encodes for bone morphogenetic protein 15 (BMP-15) which has been widely studied, especially in the context of its cooperative role with growth differentiation factor 9 (GDF-9), which is produced by the oocyte^42^. When working as a heterodimer, these factors regulate follicular cell growth and differentiation^42^. Our dataset suggests that transcription of BMP-15 is highest during the middle of culture, when the follicle is expanding, driven by the proliferation and differentiation of its somatic cells.

Current culture systems provide follicles in vitro with a “static” nutritional environment, made up of the same supplements at the same concentration throughout culture. But the growing follicular structure may need a more dynamic cocktail of factors—arriving asynchronously, to activate a properly ordered cascade of events—for optimal growth and maturation in vitro^43,44^. For example, the cyan cluster (late activators) was enriched for genes with roles in cytokine signaling and angiogenesis, including genes coding vascular endothelial growth factor (VEGF) and angiotensinogen (Supplemental Dataset S1). These factors are not only key for regulating vascular dynamics, but have also been shown to impact steroidogenesis in the antral ovarian follicle^45,46^. VEGF was also identified previously as a top factor distinguishing successful 10X follicle development from suboptimal 5X development^21^. Appropriately-timed supplementation with these pro-angiogenic factors may enhance follicle development in vitro by recapitulating *the *in vivo changes of microenvironment and promoting steroidogenesis late in culture. Supplementation with metabolites, hormone precursors, or factors promoting hormone production may also enhance follicle development or correct dysregulation of folliculogenesis in vitro with careful timing and appropriate dosage.

### Comparisons between 5 and 10X reveal pathways essential for successful follicle culture in vitro

While follicles cultured in 5X survive to the end of culture and show some growth, their growth and developmental potential is inferior when compared to the 10X condition with regards to terminal diameter and oocyte maturation (Fig. 1). Comparisons of the somatic cell transcriptomes (Fig. 3) uncovered important distinctions between the two co-culture conditions that may be leveraged to improve methods for follicle culture in the future. The gene subsets shown in Fig. 3A highlight pathways and factors with differing temporal expression in 5X and 10X. For example, the yellow subset was enriched for genes related to calcineurin-NFAT signaling. Calcineurin signaling has been characterized for its role in various developmental processes across many organ systems, and in the ovary specifically it has been implicated in modulating FSH-induced upregulation of aromatase and downstream estradiol synthesis^47^. Thus, dysregulated calcineurin signaling in the in vitro follicle microenvironment may contribute to delayed follicular expansion and insufficient estradiol synthesis, leading to suboptimal oocyte quality in 5X at the end of culture. Deficient estradiol synthesis was observed in 5X follicles by Zhou et al. in their analysis of the 5X and 10X culture systems, and may be mitigated by exogenous stimulation of estradiol production with various factors, such as using exogenous calcineurin or oocyte-secreted factors such as BMP-15 and GDF-9 known to stimulate estradiol production^47,48^. The green subset was enriched for genes related to *Wnt* signaling and angiogenesis, specifically genes coding for angiogenin proteins. Properly timed activation of angiogenesis-related signaling cascades is not only essential in the follicle’s native microenvironment for vascularization of the theca layer, but may also impact proper steroidogenesis and thus development of the antral follicle. The VEGFs and angiopoietins have been characterized in the context of ovarian follicle development, but other cytokines such as angiogenins have yet to be studied in the context of folliculogenesis. Therefore, follicle development in vitro may be enhanced by carefully timed supplementation with pro-angiogenic factors like VEGF or angiopoietin, or by experimental supplementation with angiogenins earlier in culture as evidenced by the decrease in expression of these genes over time in 10X. The cyan cluster was enriched for signaling pathways associated with lactation due to the presence of genes encoding for prolactins. The role of prolactins in ovarian follicle development has not been fully elucidated, although various studies cited the presence of these proteins in the follicular fluid in numerous species^49,50^. Thus, exogenous prolactin supplementation may improve ovarian follicle development in vitro despite the lack of knowledge on its mechanisms of action.

The genes represented in Fig. 3B showed similar temporal transcriptional trajectories in 5X and 10X and highly enriched for terms related to cell cycle, which is reflected in the rapid cell proliferation observed during in vitro follicle development. The genes shown in Fig. 3C showed enrichment for broad gene ontological processes related to G protein-coupled receptor signaling, response to chemical stimulus, and others that may be dysregulated in 5X compared to 10X but are not as dramatically different as those in Fig. 3A.

The targeted analyses of hormone synthesis, cytokine production, and transcription factor activity from our earlier study (Zhou et al. 2018), combined with the unsupervised transcriptome analysis presented here, highlight biological processes and pathways that may be targeted in a temporal manner to optimize healthy follicular growth in in vitro systems^21^. Temporal changes in the transcriptional profile observed in this study are supportive of the superior hormone synthesis, including estradiol production, we have found in the 10X system (Zhou et al. 2018) linking transcriptional changes with functional changes. Likewise, the cytokine profiles from our earlier study are in agreement with the pathways highlighted in this study, especially with regards to the importance of angiogenic factors in follicle development. With this study, we have expanded on the transcription factor profiling from Zhou et al. and have identified additional target pathways and factors for modulating follicular cell activity. The results of these two studies, as shown in Fig. 4D, together provide a data-driven basis for modulating these pathways in future studies and using the aforementioned factors to improve ovarian follicle development in vitro.

### Comparisons of in vitro to ovary-derived transcriptome data point to strengths and areas of improvement for in vitro culture systems

While analysis of culture systems continues to yield important information towards improvements of culture methods, comparing in vitro-cultured follicles to those growing in the native ovarian microenvironment may also yield important information on the strengths and shortcomings of current culture methods. By comparing our dataset from follicles cultured in 5X and 10X to follicles freshly isolated from the mouse ovary, we identified similarities and differences that may lead to improvements of in vitro culture methods. Transcriptional profiles during early folliculogenesis were similar in vivo and in vitro and commenced to diverge at a later stages of ovarian follicle development (Fig. 4B). This may be due to the immature follicle’s reliance mainly on paracrine factors provided by the other surrounding follicles, which are present in vitro to some extent. However, it is plausible that the in vitro microenvironment does not fully recapitulate the endocrine regulation in vivo that becomes essential in later stages of follicle development. While there were similar biological processes activated both in vivo and in vitro, such as cell cycle and metabolic processes, differences in signaling and responses to chemical stimulus were also observed (Fig. 4C). The lesser agreement in the activated and deactivated biological processes at later stages of follicle development allows us to develop data-driven hypotheses that can be tested in future research, such as the under activation of steroidogenesis in vitro compared with in vivo. Nevertheless, it is evident that follicles co-cultured in vitro can produce mature oocytes while in vitro culture of individual primary follicles failed to do so (Fig. 1F) and our results reproduced and thus further validated prior observations.

### Strengths and limitations

To the best of our knowledge, the results presented here constitute the first transcriptomic time-series dataset comparing insufficient (5X) and optimized (10x) follicle co-culture conditions of primary murine follicles for identifying targets to improve future in vitro culture systems*.* The timing of culture, tracking follicle development from the primary to fully mature stages, highlights the transcriptional changes in vitro and correlates these changes to phenotypic markers of maturation such as cell proliferation and antrum formation. The longitudinal study, which includes a 7-point time series for both 5X and 10X conditions, provides a detailed picture of transcriptional trajectories in culture. The untargeted genome-wide transcriptional microarray allows for the identification of numerous pathways and biological processes working in concert to drive folliculogenesis, as opposed to targeted approaches such as qPCR. The thorough analytical approach and statistics applied may also serve as a future model for analyzing microarray data with multi-point time series. Conversely, it is important to acknowledge the limitations of transcriptional microarray techniques compared to other transcriptional approaches such as quantitative PCR or next generation sequencing, such as RNA-seq or single cell RNA-seq. Single cell sequencing approaches applied to this culture system could uncover more targets and could clarify the identity and relative abundance of major somatic cell types (e.g., theca cells, cumulus and mural granulosa cells). The target factors and pathways presented in this study could be characterized further using other omics techniques that measure downstream consequences of the active signaling and metabolic pathways, such as proteomics and metabolomics profiles. Future studies evaluating the transcriptional changes of the growing oocyte, in addition to validation of these targets by modulating these pathways for individual follicle culture, will lead to an optimized individual follicle culture system for clinical translation. Moving forward, similar transcriptional approaches should be applied to comparing murine and human folliculogenesis and identifying factors driving human folliculogenesis specifically, to work towards clinical implementation of in vitro follicle culture.

### Conclusions and future work

In vitro ovarian follicle culture is an active area of research aimed towards providing survivors of childhood cancer with the opportunity to have their own biological children. Patients’ cryopreserved ovarian tissue is largely made up of primordial and primary follicles, which biology is less understood than late stage follicles and they present a significant challenge to culture in vitro*.* Improved culture methods require a deeper understanding of the mechanisms of early folliculogenesis in vivo and how in vitro culture may differ. Primary follicle co-culture is a step towards understanding these mechanisms and provides a means to achieve meiotically-competent oocytes, but this method has limited clinical translation. A universal rescue cocktail created based on transcriptional signatures identified in multiple follicle co-culture would provide the individual follicle with the proper nutrients in relevant concentrations and at the appropriate stages of development. Previous targeted secretome analysis identified a few factors that may improve individual follicle growth in vitro*.* However the transcriptome analysis presented here represents a more thorough attempt to identify novel target pathways and cell processes that may be manipulated exogenously to improve in vitro culture outcomes^21^. Additionally, comparisons between this dataset and previously published data show transcriptomic similarities and differences between follicles cultured in vitro and those isolated from the in vivo environment. Further refinement of media conditions with regards to both relevant signaling pathways in folliculogenesis and necessary metabolites may bring successful early-stage individual follicle culture closer towards clinical applications in assisted reproductive technologies.

## Materials and methods

### Approach

To identify temporal changes in upstream regulators of follicle growth and maturation that contribute to the successful culture of early follicles in vitro, microarray analysis of somatic cell samples from primary follicles co-cultured in 5X and 10X was performed.

### Follicle isolation, encapsulation, and culture

Whole mouse ovaries were enzymatically digested to harvest large numbers of primary follicles. Enzymatic follicle isolation was performed as previously described^21^. Briefly, ovaries were taken from female C57B6 X CBA/J mice ages 10–12 days. All animal procedures were performed in compliance with the Guidelines for the Care and Use of Animals at the University of Michigan and approved by the Animal Care and Use Committee at the University of Michigan (PRO00008465). After removing the surrounding tissue, ovaries were washed in warm Leibovitz’s L-15 medium (L-15) (Thermo Fisher) and transferred to a dish with pre-equilibrated alpha modification of minimum essential medium (αMEM) (Thermo Fisher) with 0.5% (v/v) PenStrep (Thermo Fisher). 10% (v/v) Liberase DH at 13 Wünsch units/mL (Sigma-Aldrich) was added to the dish of ovaries and gently mixed. Ovaries were incubated undisturbed at 37 °C for 35–45 min followed by 5 min of pipetting to break up the enzymatically-digested tissue into individual follicles. The digest was then arrested using 10% (v/v) fetal bovine serum (FBS) (Thermo Fisher).

Follicle encapsulation was performed as previously described^21^. Briefly, primary follicles with diameters ranging 90–110 µm were chosen from the digest and encapsulated in 10 µL alginate beads in either 5 (5X) or 10 (10X) follicles per bead. Beads containing follicles were dropped into a solution of 50 mM CaCl_2_ and 140 mM NaCl and allowed to crosslink for 2–3 min. Alginate beads were cultured in 96 well plates with 150 µL growth media (GM) (α-MEM supplemented with 3 mg/mL bovine serum albumin (BSA, MPBiomedicals, Irvine, CA, USA), 1 mg/mL bovine fetuin, 5 µg/mL insulin, 5 µg/mL transferrin, 5 ng/mL selenium (ITS, Sigma, St. Louis, MO, USA), and 10 mIU/mL highly purified, human-derived, follicle-stimulating hormone (FSH) (Urofollitropin, Ferring Pharmaceuticals, Saint-Prex, Switzerland) per well. Because of the manual follicle encapsulation, on average 10–20% of hydrogels will not fully encapsulate exactly 5 or 10 follicles. In this case, those samples were removed from culture and could not be used for microarray analysis. By doing so, we developed samples that reflected only a true 5X or 10X transcriptome signature, where each follicle was experiencing the same in vitro culture “experience” as the other follicles that it was pooled with. Every 48 h after encapsulation, half of the growth medium (75 µL) was exchanged for fresh medium. Follicle development was tracked by imaging follicles every 48 h before exchanging growth medium. Follicle diameter from each 48 h time point was measured using ImageJ by taking the average of two perpendicular measurements across the follicle. 5X or 10X hydrogels with any follicles that fell out of the alginate hydrogel onto the bottom of the well, or hydrogels with follicles dead at the start of culture, were excluded from analysis and microarray sample collection. Follicles were termed “dead” if the oocyte was extruded more than 50% from the follicular structure with no surrounding granulosa cells, or if the follicular structure was dark and the oocyte was not visible during imaging. Only follicles that survived to Day 12 of culture were included for growth analysis as shown in Fig. 1D. The results presented were pooled from 9 separate culture experiments, with cells from 430 follicles cultured in 10X and 380 follicles cultured in 5X collected to extract RNA. As an alternative to submitting biological replicates for microarray analysis, multiple hydrogels with follicles were pooled into one sample. Sample pooling such as the approach applied here has been widely studied as a way to reduce within-group variability and compensate for the loss of number of replicates, thus reducing cost and use of resources, without loss of statistical power^51–53^. These studies together point to the utility of pooled RNA samples as an alternative to additional replicates with minimal adverse effects to the data and downstream analysis. The variations in follicle numbers and total RNA content across samples do not impact the downstream analyses outlined below because of the amplification employed prior to microarray.

### Somatic cell isolation, RNA extraction, and microarray analysis

Follicles (n = 380 total for 5X and n = 430 total for 10X) were taken from culture for somatic cell isolation at the following time points: Days 2, 4, 6, 7, 8, 10, and 12. The number of follicles isolated for each microarray sample are outlined in Supplemental Table S1. To isolate somatic cells, alginate beads were digested by incubating with 10 IU/mL alginate lyase for 30 min. Follicles were then transferred to warm L-15 and oocytes were mechanically removed from the follicles using insulin syringes (BD 305620). The cells were centrifuged at 300 g for 5 min, supernatant was removed, and the remaining cell pellet was snap frozen in liquid nitrogen. The process of follicle encapsulation, culture, and cell isolation is visualized in Fig. 1A. RNA was extracted using the Qiagen RNEasy Micro Kit^®^ following the manufacturer’s instructions. Extracted RNA was stored at − 80 °C before being submitted to the University of Michigan’s Advanced Genomics Core. The RNA samples were analyzed on an Affymetrix Mouse Gene ST 2.1 plate using the Affymetrix GeneChip^®^ WT-Pico Kit. The chip was run on a GeneTitan Multi-Channel instrument at the Advanced Genomics Core.

### Oocyte maturation in vitro

Oocyte maturation in vitro was performed as previously described^54^. In four separate 5X and 10X culture experiments performed as outlined above, follicles were cultured for 10 to 12 days, at which point follicles with a diameter of at least 280 µm were removed from the alginate beads by incubating in 10 IU/mL alginate lyase in media (n = 45 follicles for 5X and n = 320 follicles for 10X). Most 5X follicles in culture, which did not reach this terminal size, were not subjected to IVM. This contributes to the large difference in the sample size between 5 and 10X. Individual follicles were aspirated from the culture plate, rinsed in warm media, and placed in maturation media (α-MEM supplemented with 10% fetal bovine serum, 5 ng/mL epidermal growth factor, 1.5 IU/mL human chorionic gonadotropin, and 10 mIU/mL FSH) for 20 h. After incubation, the expanded cumulus cells were removed by adding 0.03% hyaluronidase to each plate. Oocytes were aspirated and any remaining adherent granulosa cells were removed mechanically by pipetting. Oocytes were imaged at 400X and were classified as germinal vesicle/degenerate (GV/D), metaphase I (MI), or metaphase II (MII) based on their morphology. From the total follicles subjected to IVM (n = 45 follicles for 5X and n = 320 follicles for 10X), 39 (5X) and 204 (10X) oocytes were successfully isolated from their follicles to evaluate maturation state. The discrepancy between total follicles subjected to IVM (45 and 320 respectively) and number of oocytes imaged for IVM (39 and 204 respectively) is due to either (a) the oocyte being damaged or broken due to pipetting during transfer or (b) inability to locate the oocyte or dislodge it from the follicle for imaging.

### Computational methods for transcriptome data normalization and filtering

Raw CEL files were loaded into R (Version 3.6.1) using the *oligo* package^55,56^. Raw expression data was normalized using the RMA algorithm (Supplemental Figure S1), then gene expression data was filtered based on normalized intensity (> 3) and variability (standard deviation > 0.3) of expression across samples, reducing overall gene count from 41,345 to 13,313^57–61^ (Supplemental Figure S2). All gene expression data and subsets of the dataset were mean-centered for analysis and visualization unless otherwise noted. Heat maps were produced using the package *pheatmap*^62^. All gene annotation was performed using the package *AnnotationDbi*^59^.

### Principal component analysis of transcriptome data

All fourteen microarray samples (n = 13,313 genes per sample) were subjected to principal component analysis and plotted in the PC space as shown in Fig. 2A. PC1 accounts for 24% of the variance among samples while PC2 accounts for 12%. A line was plotted in the PC space to connect adjacent time points and highlight the trajectory of 5X and 10X samples over the course of follicle maturation.

### Temporal analysis of transcriptome data

The combined 13,313 gene dataset (5X and 10X samples together) was subjected to k-means clustering (k = 6) to group genes based on their temporal expression profile by combining the 5X and 10X samples (Fig. 2B). Genes in these six temporal clusters were individually subjected to differential expression analysis using *limma*, with the High and Low groups defined separately for each cluster according to the observed patterns (described in Supplemental Table S2)^40^. Differentially expressed genes (DEGs) with their associated *p* value from *limma* analysis were submitted to a directional gene ontology (GO) term analysis using *LRPath*, focusing on GO terms with 50 to 500 genes and using a *p* value cutoff of 0.05 (Supplemental Dataset S1)^46–48^. DEGs were also submitted to *GOrilla* for biological process GO term analysis, using DEGs as an unranked list of target genes (not accounting for *p* value) and all 13,313 genes as a background list, with a *p* value threshold of 0.01^49,50^ (Supplemental Dataset S2). Only genes with ontological annotation were retained from these analyses, excluding predicted genes.

### Gene filtering for trajectory smoothness and differential transcriptome patterns between 5 and 10X

To analyze genes with strong dynamic changes, one could simply select those with large variance over the 14 samples. However, a gene may show a large variance simply by having stochastic “flickers” over the 7-point time series without cohesion among adjacent points or having a systematic difference between the 5X and the 10X groups. To rank genes by their relative cohesion, or smoothness, along the temporal trajectories we implemented two metrics. The first is the Percent Variance Explained (PVE) by fitting the 7-point gene expression values (*y*) with a cubic function, $y = ax^{3} + bx^{2} + cx + d$, where *x* is coded as (− 3, − 2, − 1, 0, 1, 2, 3) and *a* − *d* are coefficients. PVE is defined as $PVE = V_{C} /V_{T}$, where *V*_*C*_ is the variance explained the cubic function fit and *V*_*T*_ is the total variance for the gene. Higher order functions (such as by adding a *x*^4^ term) would increase the potential ruggedness of the fitted curves and allow less-smooth genes to be selected. For each gene, PVE was calculated for the 5X and 10X series separately and then averaged. The second metric is a measure of how pairs of adjacent time points are more similar than pairs of more distant time points. For each gene, we calculated a 7-by-7 “distance” matrix for all pairs of absolute differences. We then defined Cohesion as $C = SSD_{A} /SSD_{{N - A}}$, where *SSD*_*A*_ is the sum of squares of the six adjacent Δ values for the gene and *SSD*_*N-A*_ is the sum of squares of the fifteen non-adjacent Δ values. PVE and C are highly correlated (not shown), as they capture similar characteristics, i.e., relatively smooth changes rather than random spikes not “supported” by adjacent time points.

To rank genes by differential patterns between the 5X and 10X series we ran Two-factor Analysis of Variance (ANOVA), where the 14 samples are arranged by a Group Factor (two levels: 5X, 10X) and a Time Factor (7 levels). We extracted the F scores for both the main effects and the interaction effect and focused on selecting those with a large F score for Interaction, F_int_. We used the three aforementioned metrics to retain genes satisfying PVE > 0.6, C < 0.35, and F > 2, narrowing the total set of 41,345 initial, unfiltered genes to 6,119 genes. This gene list of interest was clustered using a 3 × 3 self-organizing map producing 9 gene subsets with differing temporal expression patterns. These gene subsets were subjected to gene ontology analysis using *GOrilla*, filtering for gene ontology terms with less than 500 genes^32,63^.

### Comparisons to previously published transcriptome data

To compare the in vitro follicle culture data to previously published microarray data from freshly isolated follicles of different stages, raw expression data (GSE 97,902) from the Gene Expression Omnibus was downloaded using the package *GEOquery*^33,64^. This dataset reports ovarian follicle gene expression at various stages of development, using RNA collected from freshly isolated follicles from CD-1 mice^33^. From this dataset, the three replicates each of primary, multilayer secondary, and antral follicle transcriptome data were extracted for comparisons to the in vitro co-culture data. 11,424 genes were expressed in both datasets (freshly isolated follicles from GSE 97,902 or “in vivo” and somatic cells from in vitro co-culture in 5X and 10X). The datasets were normalized together using quantile normalization from the package *preprocessCore* (Supplemental Figure S4)^65^*.* To compare gene expression between the in vivo and co-culture datasets while still acknowledging the inherent differences between the datasets due to different microarray chip platforms and thus differences in raw intensity reads, the two datasets were separately subjected to DEG analysis using *limma*, with co-culture data being grouped into “Early”, “Middle”, and “Late” culture as outlined in Fig. 4A^57^. The mean expression for each gene between 5 and 10X was used as the gene expression value for comparisons to the in vivo dataset. From the DEG analysis using *limma,* each t-score was collected and used to represent an upregulation (t-score > 0) or downregulation (t-score < 0) in the gene from one stage of development to the next (Early to Middle, Middle to Late, and Early to Late). Agreement and disagreement in t-scores was visualized in Fig. 4B. Genes with t-score agreement between the in vivo and co-culture dataset were collected and subjected to *GOrilla* analysis as described above (Supplemental Dataset S4, Supplemental Image File Folder S3)^63,66–69^.

## Supplementary Information

Supplementary Information 1.

Supplementary Information 2.

Supplementary Information 3.

Supplementary Information 4.

Supplementary Information 5.

## Data Availability

We fully support rapid release of generated data and methodologies in accordance with all NIH policies. We will share with the community without restriction all protocols, experimental data, and analysis scripts. Details of ovarian follicle isolation and hydrogel encapsulation have been described in the manuscript. For microarray, the following data elements will be cleaned, annotated, packaged, and shared prior to publication: (1) Raw sequencing data will be made available at the NCBI Short Read Archive. (2) Normalized data will be provided at the Gene Expression Omnibus. (3) All data mining codes will be deposited in Github. The codes will be in R markdown language, which is good for code annotations and presentation of QC diagnostics. Each dataset created will be accompanied by an R markdown file to document the preprocessing steps, basic statistics, and profile figures.
