# Supplementary material for: Capitalizing on transcriptome profiling to optimize and identify targets for promoting early murine folliculogenesis in vitro
Source: Sci Rep. 2021 Jun 15;11:12517. doi: 10.1038/s41598-021-92036-y (PMC8206164; doi:10.1038/s41598-021-92036-y)
Supplement: Supplementary file 5 — Supplementary Information 5. [file 41598_2021_92036_MOESM5_ESM.docx]

**Supplementary Information for**

Capitalizing on transcriptome profiling to optimize and identify targets for promoting early murine folliculogenesis *in vitro*

Andrea Jones, Beatriz Peñalver Bernabé, Vasantha Padmanabhan, Jun Li, and Ariella Shikanov

Corresponding Author: Ariella Shikanov

Email: [shikanov@umich.edu](mailto:shikanov@umich.edu)

**This PDF file includes:**

Figures S1 to S4

Table S1 to S2

Legends for Image File Folders S1 to S3

Legends for Datasets S1 to S4

SI References

**Other supplementary materials for this manuscript include the following:**

Image File Folders S1 to S3

Datasets S1 to S4


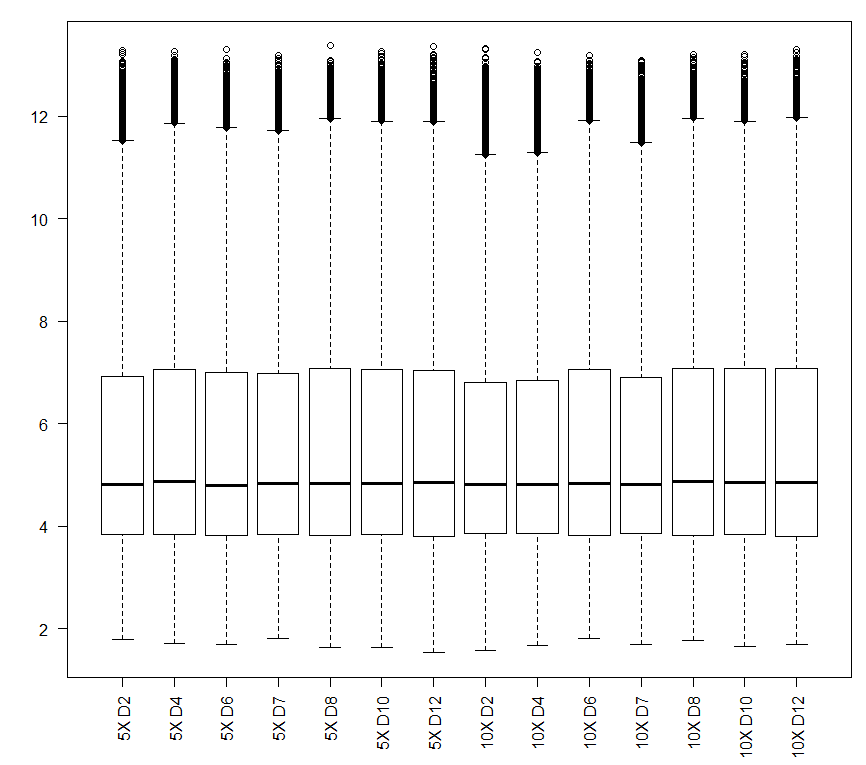


Fig. S1. Boxplot of all microarray samples after normalization. Y axis = raw intensity.


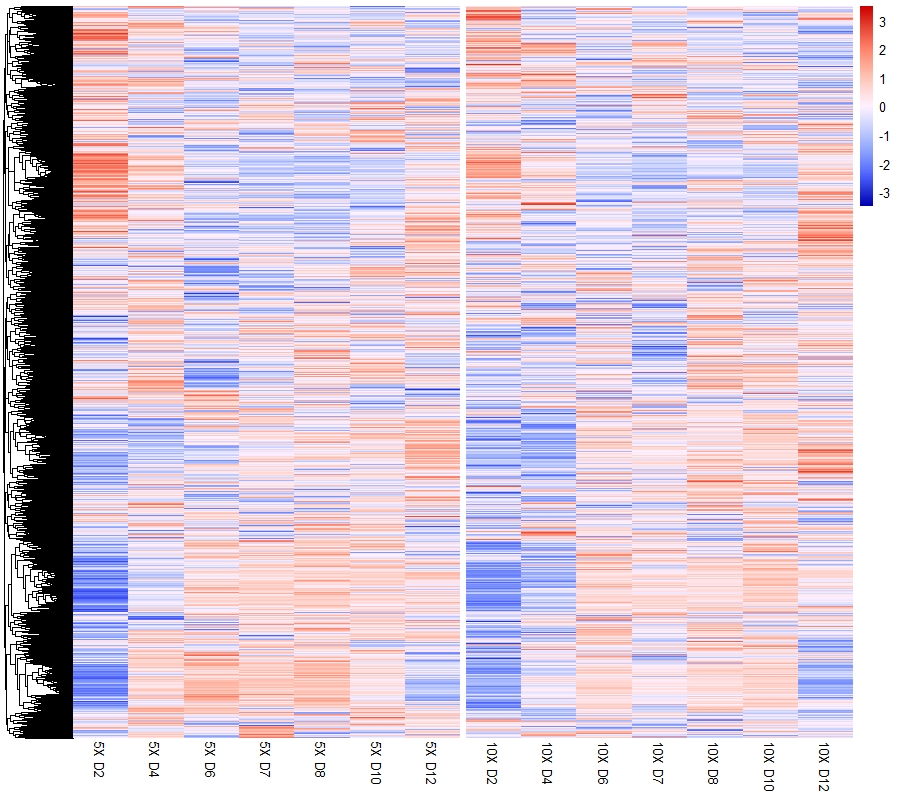


Fig. S2. High resolution heatmap of all normalized gene expression (n = 13,313 genes) across all 14 microarray samples. Plot was generated using pheatmap (version 1.0.12)^59^ in R (version 3.6.9, https://www.r-project.org/)^52^.


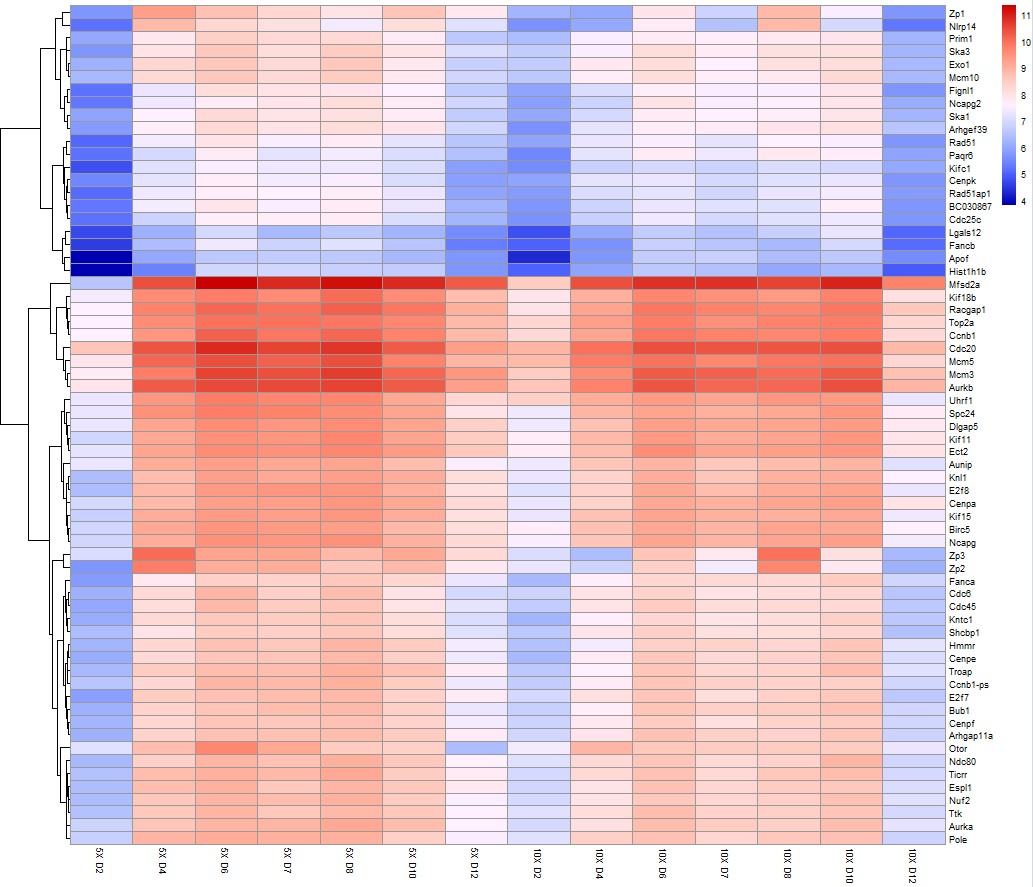

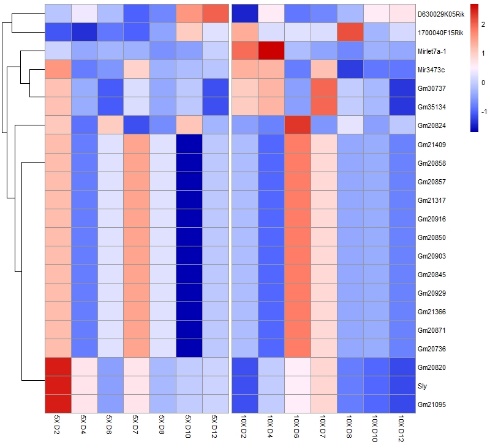
**
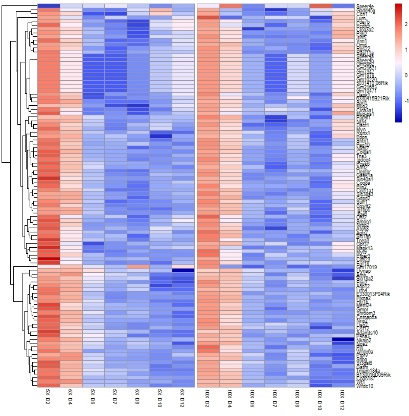

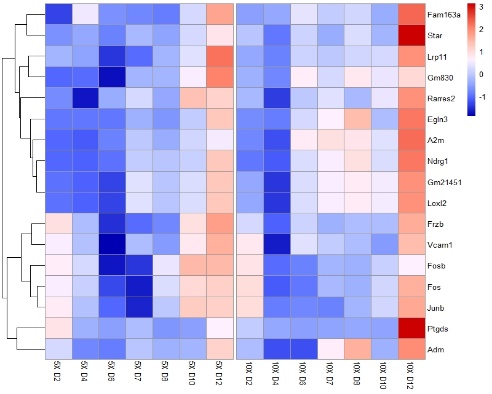
**

**B**

**A**

**C**

**D**

**E**


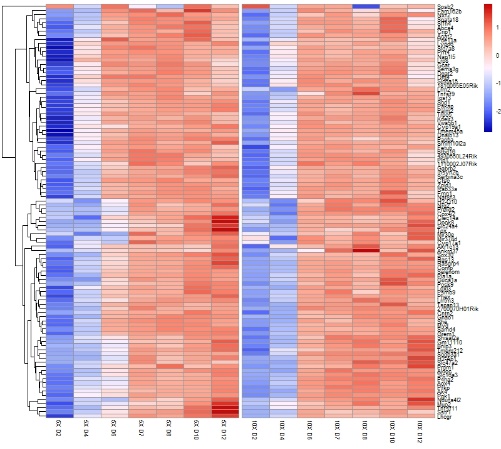


**Fig. S3.** Top differentially expressed genes from LR path analysis as reported in Figure 2. a) Top 100 genes with logFC > 1.5 for the magenta cluster, b) top 17 genes with logFC > 1.5 for the purple cluster, c) top 65 genes with logFC > 1.5 for the blue cluster, d) top 100 genes with logFC > 1.5 for the orange cluster, and e) top 17 genes with logFC > 1.5 for the cyan cluster. A maximum of 100 genes were plotted for each cluster; all genes with logFC > 1.5 were plotted for clusters with less than 100 genes meeting these criteria. Plots were generated using pheatmap (version 1.0.12)^59^ in R (version 3.6.9, https://www.r-project.org/)^52^.


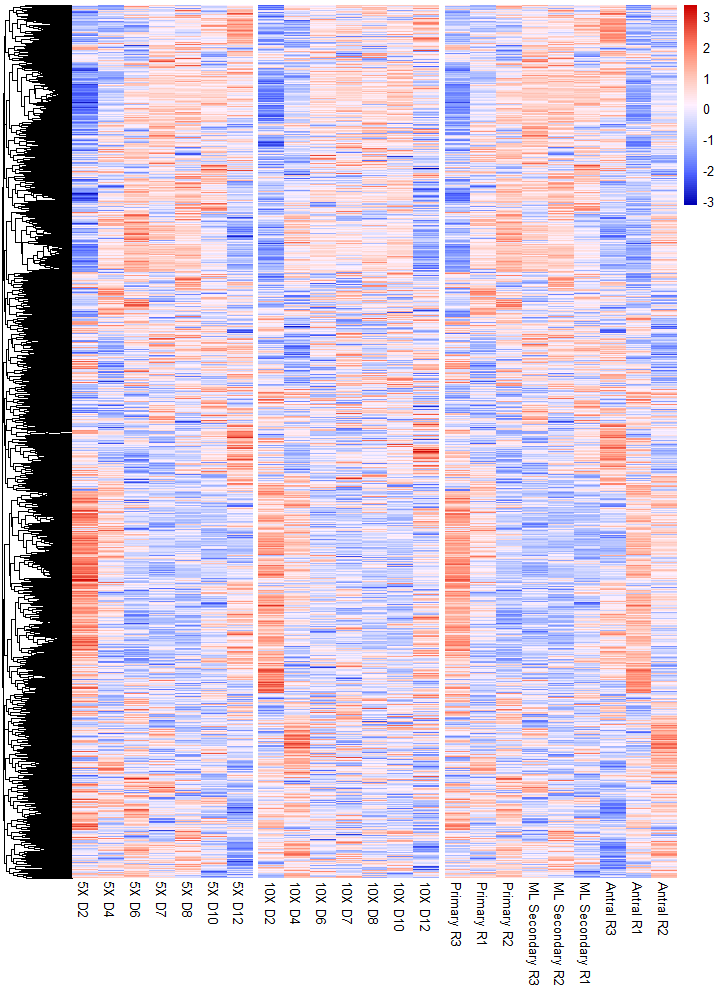


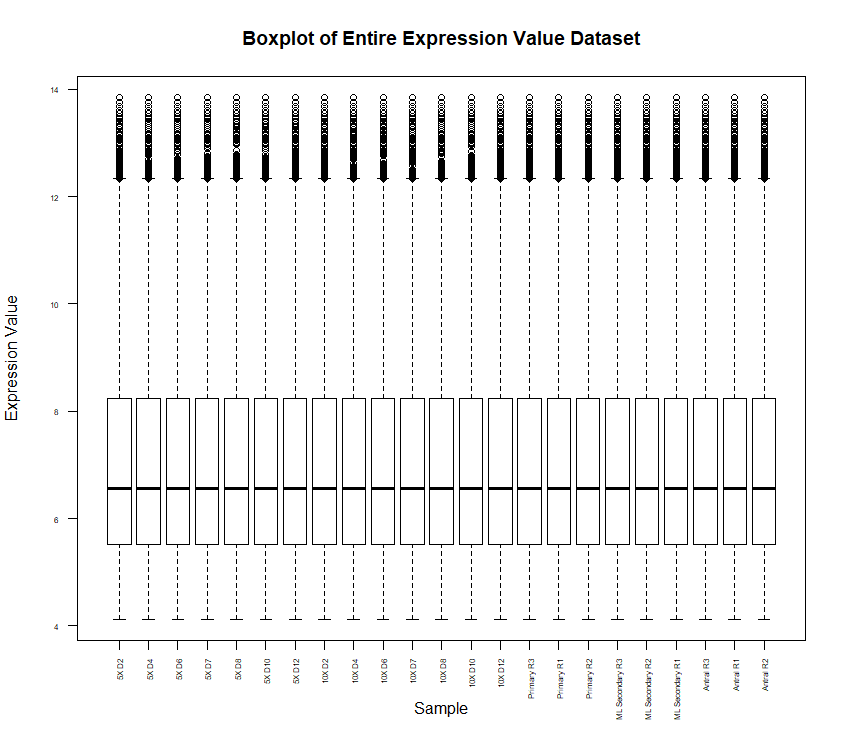


**Fig. S4.** Boxplot and heatmap of in vitro microarray samples with in vivo microarray samples, normalized together as outlined in the methods section. The heatmap shows all 11,424 genes shared between the datasets. Plots were generated using R (version 3.6.9, <https://www.r-project.org/)52>, with the heatmap on the right also using pheatmap (version 1.0.12)^59^.

| **Shading Denotes Experiment Number** | | | | | | | | |
| --- | --- | --- | --- | --- | --- | --- | --- | --- |
| 1 | 2 | 3 | 4 | 5 | 6 | 7 | 8 | 9 |

Table S1. N values for each of the 14 microarray samples.

| Cluster Color | High | Low |
| --- | --- | --- |
| Magenta | 5X Days 6 to 12, 10X Days 4 to 12 | 5X Days 2 and 4, 10X Day 2 |
| Yellow | 5X Days 2, 4, 8, 10, 12 and 10X Days 2, 4, 6, 8, 10, 12 | 5X Days 6 and 7, 10X Day 7 |
| Purple | 5X Days 4 to 12, 10X Days 6 to 12 | 5X Day 2, 10X Days 2 and 4 |
| Blue | 5X Days 4 to 10, 10X Days 4 to 10 | 5X Days 2 and 12, 10X Days 2 and 12 |
| Orange | 5X Days 6 to 12, 10X Days 6 to 12 | 5X Days 2 and 4, 10X Days 2 and 4 |
| Cyan | 5X Days 10 and 12, 10X Day 12 | 5X Days 2 to 8, 10X Days 2 to 10 |

Table S2. Table outlining experimental design for *limma* differential gene expression analysis in Figure 2; all regressions were performed high~low.

Image File Folder S1 (separate file). GOrilla charts from temporal GOrilla gene ontology analysis. The folder includes six files – one for each of the six clusters outlined in Figure 2.

Image File Folder S2 (separate file). GOrilla charts from 5X vs. 10X GOrilla gene ontology analysis. The folder includes seven files – one for each of the analyses outlined in the methods section for comparison of 5X and 10X transcriptomes.

Image File Folder S3 (separate file). GOrilla charts from *in vitro* vs. *in vivo* GOrilla gene ontology analysis. The folder includes three files – one for each of the analyses outlined in Figure 4.

Dataset S1 (separate file). Complete dataset from temporal LR Path analysis reported in Figure 2. The dataset includes six spreadsheets – one for each of the six clusters outlined in Figure 2.

Dataset S2 (separate file). Complete dataset from temporal GOrilla analysis. The dataset includes six spreadsheets – one for each of the six clusters outlined in Figure 2.

Dataset S3 (separate file). Complete dataset from 5X v. 10X GOrilla analysis. The dataset includes seven spreadsheets – one for each of the analyses outlined in the methods section for comparison of 5X and 10X transcriptomes.

Dataset S4 (separate file). Complete dataset from *in vitro* vs. *in vivo* GOrilla analysis. The dataset includes three spreadsheets – one for each of the analyses outlined in Figure 4.
